# *Streptococcus pyogenes* Pericarditis with Resultant Pulmonary Trunk Compression Secondary to Mycotic Pseudoaneurysm

**DOI:** 10.1155/2018/3514797

**Published:** 2018-08-16

**Authors:** E. Fry, J. Urbanczyk, J. Price, R. Digiovanni, M. Jepson, D. Gantt

**Affiliations:** Baylor Scott and White Medical Center, Temple, TX, USA

## Abstract

Purulent pericarditis is a rare disease in the era of antibiotics, with *Streptococcus pyogenes* being a possible, though uncommon etiology. Even more uncommon are mycotic aneurysms secondary to group A strep purulent pericarditis and bacteremia. We report a case of an 18-year-old female with a history of strep pharyngitis develop *Streptococcus pyogenes* purulent pericarditis with subsequent ventricular fibrillation (VF). Following initial stabilization, she ultimately developed a 4.8 cm mycotic aneurysm of the ascending aorta, with resultant compression of the pulmonary trunk and right pulmonary arteries.

## 1. Introduction

Purulent pericarditis is a rare but rapidly progressing and in some cases deadly event. Rapid detection via imaging and high clinical suspicion are paramount. Purulent pericarditis can, in limited cases, complicate clinical course by instigating mycotic pseudoaneurysm formation in the aorta. Proper imaging and prompt treatment with antibiotic therapy and either endovascular or open surgical repair are vital. We present a case of group A strep purulent pericarditis that resulted in formation of a mycotic pseudoaneurysm in an otherwise healthy 18-year-old female. She underwent subsequent urgent surgical repair with complete recovery. To our knowledge, this is the second reported case of purulent pericarditis complicated by mycotic pseudoaneurysm.

## 2. Case Description

An 18-year-old female with an allergy to penicillin and a past medical history of migraines presented to the emergency department with pleuritic chest pain and dyspnea on exertion. Three months prior she had an upper respiratory infection. Her initial workup was unrevealing, including negative troponin and normal complete blood count. Her electrocardiogram (ECG) revealed normal sinus rhythm. A computed tomography angiogram (CTA) chest had no significant findings. She was diagnosed with atypical chest pain and discharged home.

**Figure 1:**
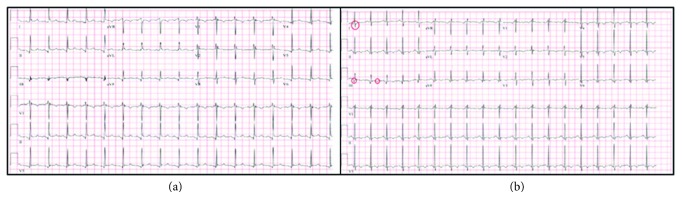
Significant ECGs. (a) Diffuse ST elevation and PR depressions indicating pericarditis. (b) S1Q3T3 phenomenon.

She returned three days later with worsening chest pain. ECG (Figure 1(a)) was significant for sinus tachycardia, PR depressions, and diffuse ST elevations, consistent with pericarditis. Significant findings at that time included a Troponin-I of 0.28 ng/ml and white blood cell count (WBC) of 16.2 (16,200). Shortly after admission, she was transferred to the intensive care unit (ICU) for hypotension and tachycardia in the 150 s. Echocardiography demonstrated a moderate pericardial effusion with evidence of tamponade. Pericardiocentesis yielded 300 ml of serous fluid and established hemodynamic stability. She was initiated on empiric antibiotic therapy with vancomycin and meropenem. Later that same day, she underwent emergent intubation and vasopressor support after two separate episodes of ventricular fibrillation and pulseless electrical activity, requiring multiple rounds of advanced cardiac life support. After stabilization, fluid aspiration from the pericardial drain revealed 130 ml of purulent fluid. Her final pericardial fluid cultures and blood cultures grew *Streptococcus pyogenes*. Antibiotic coverage was weaned to intravenous cefazolin monotherapy. She continued to drain 240–360 milliliters of purulent pericardial fluid daily and was on norepinephrine for pressure support. Post resuscitation, she developed acute renal failure that required intermittent renal replacement therapy secondary to acute tubular necrosis. Antibiotic coverage was broadened to vancomycin and cefepime after the development of acute respiratory distress syndrome (ARDS) secondary to multifocal pneumonia. She was gradually weaned from the ventilator and subsequently extubated. The remaining hospital course was uneventful, and she was discharged home on oral levofloxacin for completion of her antibiotic course.

**Figure 2:**
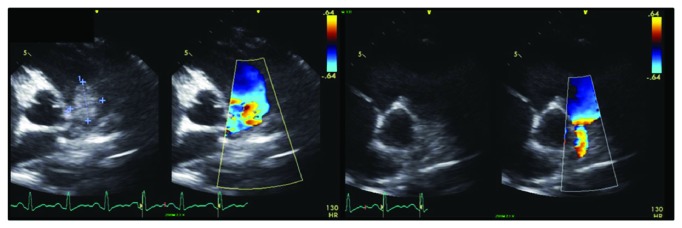
Transthoracic 2D echocardiography images. Echo images concerning for compression of the pulmonary vasculature.

**Figure 3:**
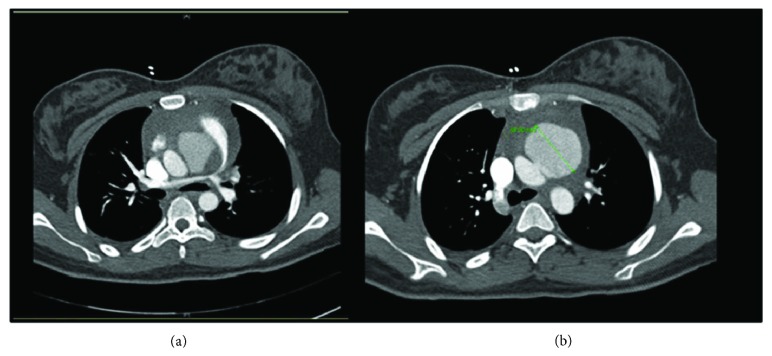
CT scans identifying pseudoaneurysm. Pseudoaneurysm compressing on the pulmonary trunk and right main stem (a) and 4.8 cm aortic pseudoaneurysm (b).

The patient returned to the ED several days later with shortness of breath and worsening back pain that radiated to her chest. The ECG (Figure 1(b)) at that time was significant for sinus tachycardia and an S1Q3T3 phenomenon. She was admitted for severe sepsis and started on ceftriaxone for concern of recurrent bacteremia. Repeat transthoracic echocardiography demonstrated right ventricular strain and what appeared to be a near to total occlusion of her pulmonary trunk (Figure 2). Computed tomography (CT) angiography was significant for a 4.8 cm small-necked pseudoaneurysm arising off the anterolateral aspect of the aorta and was found to be compressing the pulmonary trunk and right pulmonary artery (Figure 3).

**Figure 4:**
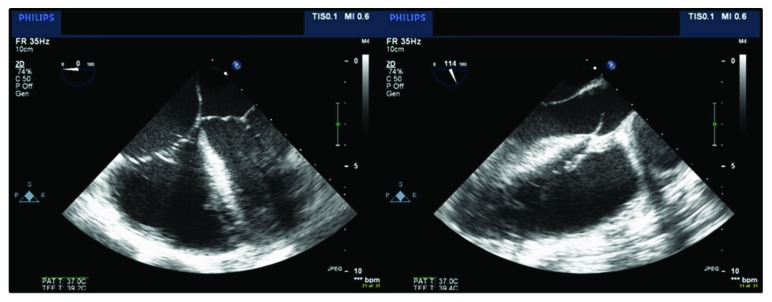
Intraoperative transesophageal echocardiogram. D-shaped ventricle indicating right heart strain seen in multiple frames.

Our patient was again transferred to the ICU. A subsequent CT chest with contrast demonstrated pseudoaneurysm expansion to 5.3 cm. Emergent cardiothoracic surgery with circulatory arrest was performed. The operation revealed a 2 cm × 1 cm wall defect in the distal ascending aorta, extending into the arch of the aorta. Intraoperative transesophageal echocardiography revealed right ventricular remodeling resulting in a severely strained D-shaped ventricle and severely dilated RVOT of 4 cm (Figure 4). Additionally, there was significant clot burden in the anterior mediastinum encasing the ascending aorta and the pulmonary artery. A CorMatrix patch was used to close the defect, and a specimen of the anterior mediastinal mass was sent for pathological analysis, confirming our diagnosis of a mycotic pseudoaneurysm. The patient tolerated the surgical procedure well and did not require additional operations during her inpatient hospital recovery.

## 3. Discussion

Purulent pericardial effusions are a relatively uncommon occurrence, particularly in the post antibiotic era. A retrospective study in Spain, done between 1972 and 1991, indicated an incidence of 33 in a population of 593,600 [1]. It is most often caused by gram positive cocci (40–45%) with *Staphylococcus aureus* comprising the majority of cases. Other commonly reported organisms include *Streptococcus pneumoniae*, *Haemophilus influenzae,* viridans streptococci, and anaerobic bacteria [2, 3]. There is a scarcity of reported group A streptococcal purulent pericardial effusions in current publications. Literature review yielded 11 previously reported cases of which only one formed a mycotic pseudoaneurysm [4]. Patients' age ranged from 4 months to 14 years old [4–12]. Dissemination of the bacteria to the pericardium typically occurs via 4 major mechanisms: (1) direct pulmonary extension such as streptococcal pneumonia/pharyngitis infection 20–25%; (2) hematogenous spread 22–29%; (3) perforating injury or surgery 24–29%; and (4) myocardial abscess and/or endocarditis in 14–22% [2, 3]. Certain factors can predispose towards their occurrence, such as transplant recipients, immunocompromised state, or chronic comorbidities. Common complications from purulent pericarditis can include constrictive pericardial disease due to thickening and scarring of the pericardium or pericardial empyema [13]. Despite treatment, up to 40% of patients die from tamponade, constriction, or toxicity [14]. Previously described successful treatments include a 2-to-4-week duration of intravenous antibiotic monotherapy with *β*-lactam agents or combination therapy with a *β*-lactam and aminoglycoside [7].

Mycotic pseudoaneuryms are rarely described following purulent pericardial effusions and only comprise a small minority of all aneurysms (0.7%) [15]. Hematogenous seeding of a damaged atherosclerotic wall is the most frequently described pathogenesis for infected aortic aneurysms [16], with staphylococcal and salmonella spp. being the most common [17]. Other predisposing factors include impaired immunity, arterial injury, or preexisting aneurysm [15, 18, 19]. Foroulis et al. and Sorensen et al. previously describe aortic aneurysms involving pulmonary artery compression, though neither was mycotic in origin [20, 21]. Management of pseudoaneurysm typically consists of antibiotic therapy and either endovascular or open repair. An endovascular approach is preferred in high-risk patient, patients with significant comorbidities, or as a bridge to future surgical repair [19, 22]. Inherently, endovascular repair carries an increased risk of secondary infections and graft failure. Open surgical repair has been shown to have an initial higher mortality rate compared with endovascular repair but displays improved long-term bacterial clearance and mortality benefit [19, 22].

## 4. Conclusion

Purulent pericarditis is a rare and serious condition with a high chance of complications and mortality. We describe a rare case of group A streptococcal purulent pericarditis with multiple complications, including mycotic pseudoaneurysm formation and resultant compression on the pulmonary vasculature. While unusual, mycotic aortic pseudoaneurysms are a reported complication following purulent pericardial infection. Appropriate and early antibiotic therapy was key in our patient's recovery as well as swift pseudoaneurysm identification and surgical intervention.
